# Functional Vitamin B12 Deficiency in Association With Nitrous Oxide Inhalation

**DOI:** 10.7759/cureus.21394

**Published:** 2022-01-18

**Authors:** Elisabetta Porruvecchio, Sophiya Shrestha, Bao Khuu, Usman Iqbal Rana, Maaryah Zafar, Mansoor Zafar, Amarah Kiani, Abubakar Hadid

**Affiliations:** 1 Internal Medicine, Conquest Hospital, East Sussex Healthcare NHS Trust, St. Leonards-on-Sea, GBR; 2 General Internal Medicine, Conquest Hospital, East Sussex Healthcare NHS Trust, St. Leonards-on-Sea, GBR; 3 Medicine, Universitatea din Oradea, Oradea, ROU; 4 Gastroenterology, Hepatobiliary, and Hepatology, Conquest Hospital, East Sussex Healthcare NHS Trust, St. Leonards-on-Sea, GBR; 5 Radiology, Conquest Hospital, East Sussex Healthcare NHS Trust, St. Leonards-on-Sea, GBR

**Keywords:** subacute combined degeneration of the spinal cord, computed tomography (ct) imaging, mri images, nitrous oxide myelopathy, inhaled nitrous oxide

## Abstract

There is a rise in the use and abuse of nitrous oxide (N₂O) as a recreational drug. In spite of the laws enforced internationally, it remains readily available and is an inexpensive mode of recreational drug. Commonly known as the ‘laughing gas’, its use as a euphoric agent is on the rise. Subsequently, the side effects are also coming to light, associated with medical, financial and social implications. It is not detected in routine drug testing. The key differential in an acute setting is often confused with the query for Guillain-Barré syndrome, chronic inflammatory demyelinating polyneuropathy and malabsorption syndromes associated with vitamin B12 and folate deficiencies. This is a case report of a 21-year-old male who presented to the hospital with concerns for weakness and tingling sensations in his extremities accompanied by an inability to bear weight, which he suggested to seem to be worsening over a period of three weeks. His blood tests suggest mild deficiencies of vitamin B12 and folate levels, and MRI revealed subacute combined degeneration of the cervical cord from C2 to C6. The radiologist advised to rule out B12 deficiency and the use of nitrous oxide as a recreational drug. This led to the discussion with the patient, during which he admitted to the use of nitrous oxide. Prompt management with B12 injections intramuscularly every two to three days a week for 11 days followed by folate supplements and monthly B12 injections were advised. He was also reviewed by the physiotherapy teams, and the patient agreed to not use nitrous oxide in the future as a recreational drug.

## Introduction

The recreational abuse of nitrous oxide (N₂O) or laughing gas continues to increase in the community. It is a cheaper, euphoria-inducing inhalant agent undetectable on routine drug screening. Although previously thought to be safe, it is now known that nitrous oxide has adverse effects on multiple organ systems, including neurological, immunological and haematological systems [1]. Nitrous oxide abuse is illegal in the UK under the Psychoactive Substances Act of 2016. The prevalence of nitrous oxide use is 2.3% in England and Wales, with the highest prevalence of 8.8% in the 16-24 age group [2]. Approximately 36.8% of people in the UK will be using nitrous oxide in their lifetime [3]. From 2008 to 2017, nitrous oxide caused 28 deaths in England and Wales, mainly secondary to asphyxia [4]. In the USA, the prevalence is reported to be 2% for adolescents in the 2000-2001 National Household Survey on Drug Abuse [5].

We present a case of a 21-year-old male who has a history of inhalational abuse of nitrous oxide and presented inability to get out of the bed with brisk appearance of symptoms of subacute combined degeneration of the spinal cord one day after his shoulder arthroplasty surgery.

## Case presentation

**Figure 1:**
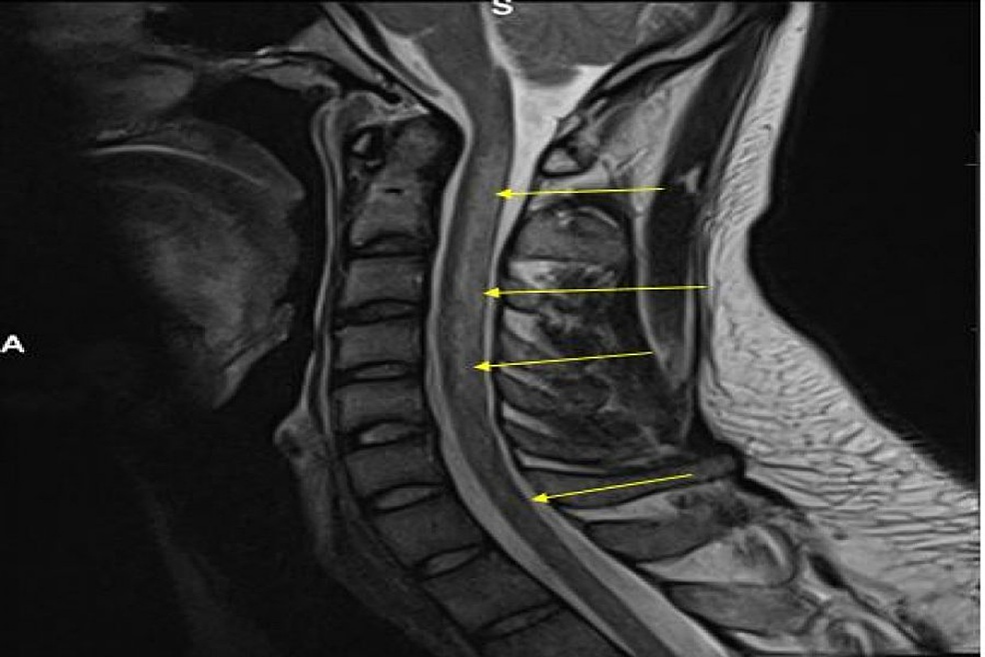
Sagittal MRI T2-weighted image showing cervical cord oedema and minimal expansion with a long segment signal abnormality discretely involving the posterior column tracts. Abnormal signal is extending up to the level of T3 (yellow arrows).

**Figure 2:**
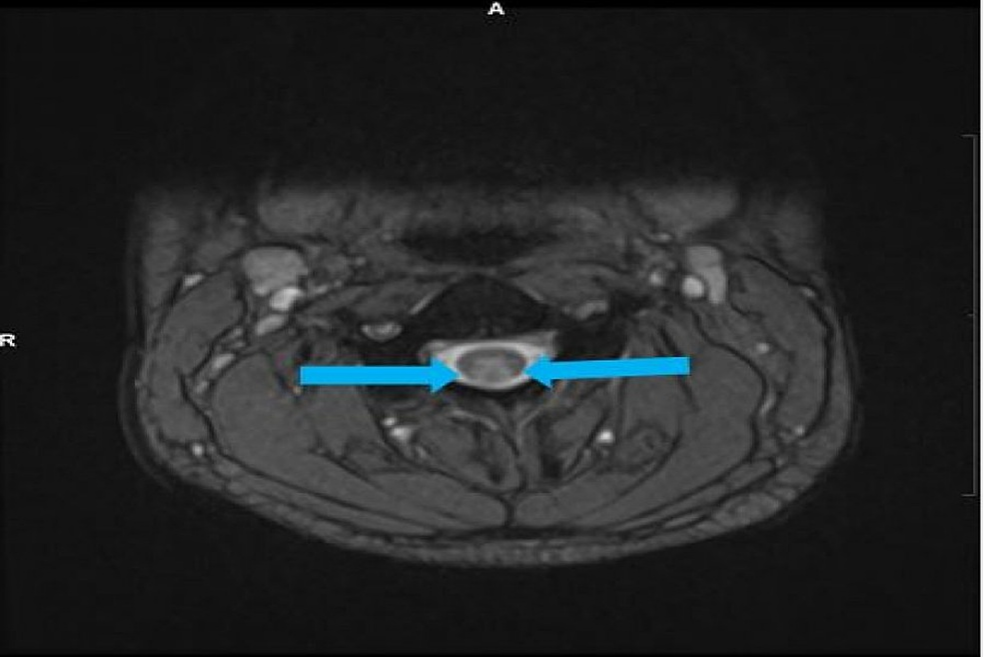
Axial MRI T2-weighted image showing abnormal bilateral high T2 cord signal in the dorsal columns over a relatively long length. The location of the signal abnormality and the length of the cord involvement are consistent with subacute combined degeneration of the cord (blue solid arrows).

A 21-year-old male presented to the emergency department and was referred to the medical team on call for concerns of three weeks history of progressively worsening weakness in his arms and legs, inability to stand on his feet, and sensations of tingling in all of his extremities. His past history included COVID-19 AstraZeneca (ChAdOx1 S (recombinant)) vaccine nine months ago, followed by the second dose three months ago. Additionally, he had a remote history of epilepsy stable while on lamotrigine. On further inquiry, he had mild symptoms of tingling in both upper and lower limbs and underwent right shoulder arthroplasty for his history of recurrent right shoulder dislocation. He was discharged home the same day post-surgery. The very next day, he was unable to get out of the bed by himself and decided to come to the hospital for further review. Further discussions with the anaesthesia department revealed that nitrous oxide was not used during the orthopaedic management of the right shoulder dislocation. On examination, he was afebrile and normotensive. Chest, cardiovascular and abdominal examinations were inconclusive. Neurological examination revealed normal reflexes in the upper limbs with brisk knee reflexes and positive Babinski in both lower limbs. His lower limb position and vibration sensations were impaired, along with impaired sensation to touch that normalised above the knees bilaterally. Motor examination showed power 4/5 bilaterally in the upper limbs and 3/5 in the lower limbs. Romberg was positive. The cerebellar examination was normal. MRI of his cervical spine with T1- and T2-weighted images showed sagittal T1 and T2 and STIR sequences. There was a long segment cord signal abnormality involving the dorsal columns with low T1 and high T2 signals extending from C2 to C6 level. Vertebral body heights and intervertebral disc spaces were preserved. There was no focal marrow signal abnormality, nor there was any evidence of spinal canal stenosis (Figure 1, 2).

The radiologist suggested considering differentials of subacute combined degeneration of the cord (vitamin B12 deficiency), nutritional/metabolic deficiencies (e.g., copper/vitamin E) and substance abuse (e.g., nitrous oxide poisoning). On further discussions with the patient, he admitted to using approximately 40-120 whipped cream chargers weekly. He underwent thorough blood investigations. The results suggested functional vitamin B12 deficiency with slightly low levels of vitamin B12 and high levels of methylmalonic acid and plasma homocysteine levels (Table 1).

**Table 1:** Serum markers and electrolyte levels. Source: Microbiology Laboratory, Conquest Hospital, East Sussex Healthcare NHS Trust

Investigation	Units of measurements	Normal range	Day 1	Day 5
Haemoglobin (Hb)	g/L	130–180	138	130
Mean corpuscular volume (MCV)	fL	80–100	89.3	89.7
Red cell distribution width (RCDW)	%	11.8–14.8	15.2	15.1
Erythrocyte sedimentation rate	mm/hour	1–10	-	7
Cyanocobalamin level (vitamin B12)	ng/L	197–771	112	
Methylmalonic acid (MMA) levels	nmol/L	0–280	-	699
Folate levels	ug/L	2.4–17.5	2.1	
Ferritin levels	ug/L	30–400	-	202
Homocysteine levels	umol/L	0–150	-	30.1
Thyroid-stimulating hormone (TSH) levels	mU/L	197–771	0.63	0.88
Tissue transglutaminase (TTG) levels	U/mL	0–7	0.6	-
Serum C3	g/L	0.90–1.80	-	1.42
Serum C4	g/L	0.10–0.40	-	0.31
HbA1c levels	mmol/mol	21–41	30	
Antinuclear antibody	-	-	Negative	-
Anti-myeloperoxidase antibodies	IU/mL	0–3.4	-	<0.2
Anti-proteinase 3	IU/mL	0–1.9		<0.2
Corrected calcium	mmol/L	2.20–2.60	2.20	-
Serum magnesium	mmol/L	0.7–1.0	0.77	-
Serum inorganic phosphate	mmol/L	0.8–1.5	1.06	-
Serum sodium	mmol/L	133–146	138	139
Serum potassium	mmol/L	3.5–5.3	3.9	5.1
Serum urea	mmol/L	2.5–7.8	5.7	4.3
Serum creatinine	umol/L	59–104	74	72

His serum immunoglobulin levels, thyroid-stimulating hormone levels, serum iron studies, anti-parietal cell antibodies, anti-intrinsic factor antibody, serum anti-thyroid peroxidase (TPO) antibodies, syphilis, complete viral hepatitis, HIV screening, and cerebrospinal fluid analysis including viral PCR studies were all completely normal. His lamotrigine levels in serum were also normal. A diagnosis of nitrous oxide-related subacute combined degeneration of the cord was made. Prompt management with B12 injections intramuscularly every two to three days a week for 11 days followed by folate supplements and monthly B12 injections were advised. He was also reviewed by the physiotherapy teams and underwent graded exercises to improve mobility. The patient agreed not to use nitrous oxide in the future as a recreational drug.

## Discussion

Nitrous oxide (N_2_O) has long been used as an anaesthetic, commonly used to induce general anaesthesia towards general and other surgeries. However, it is also commonly used for recreational purposes especially in the younger age group [6]. It has a tendency to cause neurotoxicity by way of interfering in the bioavailability of vitamin B12 if abused. Rarely, a fatality has also been reported [7]. Nitrous oxide is commonly available as ‘whippet’ canisters. It is used to fill balloons or whipped cream dispensers by recreational users and inhaled from balloons or the dispenser for recreational purposes [8].

The mode of subacute combined degeneration in the setting of N_2_O abuse appears to be mediated by B12 deficiency, which is functional, resulting in decreased normal myelin synthesis. A metabolite of cobalamin, namely, 5-deoxyadenosylcobalamin, a coenzyme catalysing the conversion of methylmalonyl CoA to succinyl CoA, which subsequently enters the citric acid cycle, is compromised. Nitrous oxide oxidises the cobalt ion of vitamin B12, which irreversibly inactivates cobalamin, which is unable to function as a coenzyme. This results in the accumulation of methylmalonyl CoA, which enters into the lipid pathway and results in the incorporation of abnormal fatty acids into neuronal lipids. The outcome is subacute combined degeneration of the spinal cord, more appropriately suggesting the decreased myelination of the lateral and posterior columns of the spinal cord [9,10].

In a separate pathway, nitrous oxide neurotoxicity is mediated through nitrous oxide binding to and inactivating cobalamin, reducing cobalamin’s effect as a cofactor in the conversion of homocysteine to methionine [11]. Since methionine metabolite S-adenosyl-methionine is required for myelin synthesis and maintenance, the depletion causes neuropathy and myelopathy [12].

Subacute combined degeneration of the spinal cord usually presents with ascending paraesthesia, weakness, ataxia, and loss of sphincter control most commonly reported with vitamin B12 deficiency. MRI shows symmetric increased T2 signal in the posterior and lateral columns of the cervical and thoracic spinal cord with inverted ‘V’ sign [13].

Although methionine supplementation has some evidence of efficacy, its use is hampered by supply and availability at times [13]. Despite treatment, neurological symptoms can take months to resolve, and many patients report residual symptoms. Despite the availability of treatment, the recovery often takes months, with many patients reporting some residual symptoms [13]. Treatment consists of abstinence from the abuse of nitrous oxide and replacement of vitamin B12 [14].

## Conclusions

In a patient presenting with symptoms and signs of vitamin B12 deficiency along with low levels of vitamin B12 and folate levels, it is important to take a thorough history. In the case of low or low normal levels of vitamin B12, it is important to have plasma homocysteine and methylmalonic acid levels to confirm the diagnosis. A prompt vitamin B12 injection along with physiotherapy support is vital towards the clinical recovery albeit with residual symptoms.
